# Highly efficient red OLEDs using DCJTB as the dopant and delayed fluorescent exciplex as the host

**DOI:** 10.1038/srep10697

**Published:** 2015-05-29

**Authors:** Bo Zhao, Tianyou Zhang, Bei Chu, Wenlian Li, Zisheng Su, Hairuo Wu, Xingwu Yan, Fangming Jin, Yuan Gao, Chengyuan Liu

**Affiliations:** 1 State Key Laboratory of Luminescence and Applications, Changchun Institute of Optics, Fine Mechanics, and Physics, Chinese Academy of Sciences, Changchun 130033, People’s Republic of China; 2 University of Chinese Academy of Sciences, Beijing 100039, People’s Republic of China

## Abstract

In this manuscript, we demonstrated a highly efficient DCJTB emission with delayed fluorescent exciplex TCTA:3P-T2T as the host. For the 1.0% DCJTB doped concentration, a maximum luminance, current efficiency, power efficiency and EQE of 22,767 cd m^−2^, 22.7 cd A^−1^, 21.5 lm W^−1^ and 10.15% were achieved, respectively. The device performance is the best compared to either red OLEDs with traditional fluorescent emitter or traditional red phosphor of Ir(piq)_3_ doped into CBP host. The extraction of so high efficiency can be explained as the efficient triplet excitons up-conversion of TCTA:3P-T2T and the energy transfer from exciplex host singlet state to DCJTB singlet state.

Organic light emitting diodes (OLEDs) have being attracted tremendous attention due to their promising applications in flat-panel displays, solid state lighting and so on. In general, the ratio of singlet and triplet excitons is 1:3 under an electric excitation^1^,^2^ and the external quantum efficiency (EQE) of the traditional fluorescent OLEDs can be only limited by 5% supposing the coupled output efficiency is 20%. But recently, a new mechanism is presented to improve fluorescent efficiency, which is referred to as thermally activated delayed fluorescence (TADF). In this way, the theoretical limit of 5% is broken^3^,^4^ and the EQE can reach up to ~20%^5^,^6^. At the same time, delayed fluorescent exciplex is also developing rapidly, many highly efficient delayed fluorescent exciplexes have been reported in the last few years^7^,^8^,^9^,^10^.

OLEDs with exciplex as the host have also been demonstrated in recent years. The exciplex host behaves bipolar feature and carrier mobility can be tuned by adjusting the ratio of donor to acceptor. Further more, the carrier injection barriers that from the transport layer to emitting layer (EML) can be eliminated almost. Efficient singlet and triplet energy transfer from exciplex host to dopant can also be realized because of the presence of almost identical singlet and triplet energy levels of the exciplex. Kim and coworkers had fabricated a series of phosphorescent OLEDs by utilizing the exciplex host system^11^,^12^,^13^,^14^, which processed a low bias, high efficiency and low efficiency roll-off. But the phosphorescent materials contain the rare heavy metal, such as iridium and platinum, thus they must suffer from the high material cost and face the lack of resource in the future. Zhang and co-workers demonstrated high efficiency fluorescent OLEDs using sensitizing hosts with a small singlet-triplet exchange energy^15^. Such a host using TADF material, however, is limited and the troublesome chemical synthesis would be required. Besides, the yellow fluorescent dopant of 3,11-Diphenylamino-7, 14-diphenylacenaphtho [1,2-k]fluoranthene (DDAF) used in that paper is not an common fluorescent material and the yellow color is also not a primary color in flat-panel display or lighting applications.

It is well known that red color is one of the three primary colors (red, green and blue) in the full color displays and white lighting. Several red materials have been developed, such as both fluorescent emitter of 4-(dicyanomethylene)-2-t-butyl-6-(1,1,7,7-tetramethyljulolidyl-9-enyl)-4H-pyran (DCJTB) and phosphor material of tris(1-phenylisoquinoline) iridium(III) (Ir(piq)_3_) are the most common red emission materials. Liu and coworkers achieved the emission of DCJTB with a maximum current efficiency of 4.44 cd A^−1^ by doping 2% DCJTB into bipolar mixed host, which comprised of tris(8-hydroxyquinolinato) aluminum(III) (Alq) and rubrene^16^. Similarly, Lee *et al* fabricated red OLEDs adopting the co-host of Alq: 2-methyl-9,10-di(2-napthyl)anthracene (MADN) and the red emitter of DCJTB^17^, which offered a maximum current efficiency of 5.42 cd A^−1^ at 20 mA cm^−2^. But such low efficiency could meet the industrial requirements difficultly. The red phosphor of Ir(piq)_3_ could realize high efficiency, but the high cost due to the presence of rare heavy metal iridium and the serious efficiency roll-off at high current density are disadvantage to practical application. Chou *et al* synthesized bis-4-(N-carbazolyl)phenyl)phenylphosphine oxide (BCPO)^18^ with bipolar property to used as the host of Ir(piq)_3_, the device provided a peak EQE of 17.0% and a maximum current efficiency of 19.4 cd A^−1^, respectively. But it suffered from a serious efficiency roll-off, which dropped 45% from the practical application luminance of 1,000 cd m^−2^ to 10,000 cd m^−2^. Fukagawa *et al*^19^ employed common 4,4′-N,N′-dicarbazole-biphenyl (CBP) as the host of Ir(piq)_3_, but the device only offered a low power efficiency of 4.2 lm W^−1^ at 1000 cd m^−2^. Taking into account of the development of the delayed fluorescent exciplex, we provided a new strategy to achieve highly efficient red OLEDs by combining the delayed fluorescent exciplex with traditional fluorescent red material. That is, exciplex that performed delayed fluorescent feature as the host and red fluorescent material of DCJTB as the dopant. The singlet excitons of exciplex host are transferred directly to singlet state of the fluorescent dopant via Förster energy transfer mechanism. The triplet excitons of exciplex are up-converted to its singlet state by reverse intersystem crossing (RISC) process and then transferred to the singlet state of the dopant. At last, all the excitons could contribute to the fluorescent emission arising from the singlet state of the dopant. Figure 1 depicts the expected schematic level diagram and energy transfer process. Based on the energy transfer processes described above, the traditional fluorescent material could break the limit of maximum EQE of 5%.

In this paper, we selected the highly efficient delayed fluorescent exciplex of 4,4′,4″-tri(N-carbazolyl)triphenylamine:2,4,6-tris(3-(1H-pyrazol-1-yl)phenyl)-1,3,5-triazine (TCTA:3P-T2T)^20^ as the host and DCJTB mentioned above as the dopant. A maximum luminance, current efficiency, power efficiency and EQE of 22,767 cd m^−2^, 22.7 cd A^−1^, 21.5 lm W^−1^ and 10.15% for the red fluorescent OLEDs were achieved, respectively. Meanwhile, a relative lower efficiency roll-off of 32% between 1000 cd m^−2^ to 10,000 cd m^−2^ was earned compared to red phosphorescent OLEDs, such as, reported by Ref.^18^. This is the best result based on resemble DCJTB emission spectra to our best knowledge. Our red OLEDs with DCJTB emitter combine the advantage of high electroluminescence (EL) efficiency, low cost without rare heavy metal iridium and a lower efficiency roll-off compared to red phosphorescent OLEDs. Therefore, the red fluorescent OLEDs in this paper could satisfy the requirement of practical application.

The exciplex of TCTA:3P-T2T is a highly efficient yellow delayed fluorescent emitter with a maximum EQE of 7.8%^20^. The PL peak of TCTA:3P-T2T (1:1) lies at ~538 nm, which occurs red-shift as compared to that of both neat TCTA and 3P-T2T films. The PL spectrum of co-deposited film of TCTA:3P-T2T and absorption spectrum of DCJTB are displayed in Fig. 2. We can see that the overlap between the absorption spectrum of DCJTB and the emission spectrum of TCTA:3P-T2T is very large, which indicates an efficient resonant energy transfer from the exciplex host to DCJTB by Förster mechanism. On account of the two reasons of high efficiency with delayed fluorescent emitter and large spectral overlap, TCTA:3P-T2T exciplex was selected as the host of the fluorescent dopant DCJTB.

The structures of fabricated red OLEDs are ITO/MoO_3_ (3 nm)/NPB (20 nm)/TCTA (8 nm)/TCTA:3P-T2T (1:1): x wt% DCJTB (15 nm)/3P-T2T (45 nm)/LiF (1 nm)/Al, where x = 0.5, 1 and 1.5, and the corresponding devices are referred to as Device A, B and C, respectively. Here, NPB is N,N′-bis-(1-naphthl)-diphenyl-1,1′-biphenyl-4,4′-diamine. Figure 3 shows the schematic energy level diagram of the red OLEDs. We can see that the holes and electrons are injected from TCTA and 3P-T2T to EML without any energy barrier, respectively. The emission of DCJTB may come from the energy transfer of exciplex (TCTA:3P-T2T) or direct recombination between the trapped hole and electron on the DCJTB emitter. Figure 4 plots the EL characteristic curve with current density, luminance, current efficiency, power efficiency, EQE and EL spectra of Device A, B and C. Three devices exhibit almost an identical turn-on voltage of ~2.25 V. Such a low turn-on voltage indicates that the emission of DCJTB derives from the energy transfer of the exciplex host^21^. All the three devices offer a surprising EQE of above 5% in spite of the different doped concentrations, which break the EQE upper limit of fluorescent OLEDs. Even Device B reach a rather high EQE of 10.15%, which is the best red result based on traditional fluorescent material.

From Fig. 4, we also find that the EL efficiency and spectra are very sensitive to the DCJTB concentration. On one hand, the EL efficiency shows large difference with the changed concentrations. Device B with 1.0% concentration exhibits the maximum EQE of 10.15%, which increases by 1.36 and 1.8 times compared to Device A (0.5% concentration, EQE of 7.48%) and Device C (1.5% concentration, EQE of 5.63%), respectively. Differing from the un-doped system, the EL emission from guest material in host:guest system is from two ways: one way is the energy transfer from host to guest^22^ and another is the direct hole-electron recombination on guest molecule^23^,^24^. Let us see the energy level diagram in Fig. 3. DCJTB has a deep lowest unoccupied molecular orbital (LUMO) energy level of 3.2 eV and shallow highest occupied molecular orbital (HOMO) energy level of 5.4 eV, respectively^25^,^26^. The large difference between the HOMO energy level of donor (TCTA) and DCJTB (ΔE_HOMO_ = 0.4 eV) makes DCJTB act as a deep trap site of hole. The relative small difference between LUMO energy level of acceptor (3P-T2T) and DCJTB (ΔE_LUMO_ = 0.2 eV) applies DCJTB as a shallow trap site of electron. Thus, there exists partial guest emission from trapped charges recombination. As the DCJTB concentration increases, the trapped charges also increase and the aggregate formation at high concentration provides non-radiative recombination sites, which can efficiently quench excitons through exciton-polaron annihilation (EPA)^27^. In addition to this, as the concentration increases, the triplet-triplet energy transfer between exciplex and DCJTB via Dexter process becomes efficient^28^, which is also a loss and should be prevented. Therefore, Devivce C with the highest concentration of 1.5% among the three devices shows the lowest efficiency due to the EPA and Dexter energy transfer process. We also measured the PLQY of TCTA:3P-T2T and TCTA:3P-T2T:x% DCJTB (x = 0.5, 1.0 and 1.5). The ratio of TCTA and 3P-T2T is 1:1 in all the films. The pure exciplex has the lowest PLQY of 41.6 ± 3%. And the PLQYs of doped DCJTB films with the concentration of 0.5%, 1.0% and 1.5% are 53.4 ± 2%, 68.5 ± 2% and 61.9 ± 2%, respectively. The relative low efficiency of Device A is attributed to the low PLQY, because the strong exciplex emission is observed in Device A, which is showed in Fig. 4d. From the PLQY measurement result, we also find the Device B has the highest value. Besides the high PLQY, the relative low concentration (1.0%) suppresses the trap of a large numbers of charges, so the energy transfer between the triplet state of exciplex and DCJTB could be prevented due to the increased intermolecular distance. Thus, Device B with the optimal concentration of 1.0% earns the highest efficiency. Table 1 summarizes the performance of Device A, B and C. On the other hand, the emission peaks are different for the three devices under the different concentrations of 0.5% (586 nm), 1.0% (605 nm) and 1.5% (610 nm). Figure 4d shows the variation of EL spectra under the different DCJTB concentration. We can see that with the increase of concentration, the red-shifted spectra are observed and the exciplex emission is gradually dropped and disappeared finally, i.e., the EL spectral shape is get close to the intrinsic emission of DCJTB with increased concentration. The decline and disappearance of exciplex emission as the concentration increases also confirm the energy transfer from exciplex host to DCJTB.

In order to make clear the energy transfer mechanism and high EL efficiency further, we carried out the transient PL decay measurement. Figure 5 shows the PL spectra and transient PL decay curves of TCTA:3P-T2T and DCJTB doped into TCTA:3P-T2T with various concentrations (0.5%, 1.0% and 1.5%). Figure 5a depicts the PL spectra. To observe clearly the weak exciplex emission, we employ the logarithmic coordinates with Y axis. As Fig. 5a shows, DCJTB is the main emission band when DCJTB doped into the exciplex and the red-shift also occurs as the concentration increases. Meanwhile, as the concentration increases, the emission of exciplex decreases even disappears at the concentration of 1.5%. This demonstrates again the energy transfer from exciplex host to DCJTB. Figure 5b exhibits the transient PL decay curves. As showed in PL spectra, Fig. 5a, the monitor of peaks are 538 nm, 590 nm, 608 nm and 616 nm with the transient PL decay measurement, respectively. As expected, the exciplex of TCTA:3P-T2T exhibits two fluorescent components with prompt of 52.6 ns and delayed of 1.20 μs, respectively. But to our surprised, the emissions of DCJTB that doped into TCTA:3P-T2T exhibit similar behavior and also have two components of prompt and delayed fluorescence. In general, the fluorescent material of DCJTB only has a prompt intrinsic transient lifetime of several nanoseconds^29^. But in this work, DCJTB displays the delayed lifetime of several hundred nanoseconds, even microsecond order. Compared to previous reports, the longer transient lifetime of DCJTB in this paper indicates the delayed lifetime derives from the up-conversion of exciplex host triplet excitons, not from the triplet-triplet annihilation (TTA). The prompt and delayed lifetimes of DCJTB with different doped concentration have been added to Fig. 5b. We can see that the delayed lifetimes have a large range when DCJTB with different concentrations doped into TCTA:3P-T2T. The delayed lifetime decreases gradually as the concentration of DCJTB increases. Under optical excitation, the triplet excitons of exciplex are produced by efficient intersystem crossing (ISC) process, and the delayed component of DCJTB is resulted from the energy transfer of up-converted exciplex triplet excitons. As the concentration increases, the ISC efficiency of exciplex reduces and the RISC efficiency also reduces, which result in the delayed lifetime decreases. The delayed lifetime of exciplex also demonstrate there exist a small energy difference (ΔE_(S-T)_) between its singlet and triplet excited states, which result in an efficient RISC. So under electric excitation, the 25% singlet excitons produced on exciplex host would transfer to the singlet state of DCJTB by Förster process and the 75% triplet excitons would up-convert to singlet state through efficient RISC, then the new singlet excitons formed through up-conversion would also transfer to the singlet state of DCJTB. Therefore, highly efficient DCJTB emission could be achieved. Hence, based on the discussion above, the consistent transient decay behavior of exciplex and DCJTB evidences the emission of DCJTB is derived from the energy transfer of exciplex singlet excitons and up-converted triplet excitons.

We also fabricated the Alq host device with the structure as follows: ITO/MoO_3_ (3 nm)/NPB (20 nm)/Alq: 1.0% DCJTB (15 nm)/Alq (45 nm)/LiF (1 nm)/Al, which is referred as Device D. An interesting phenomenon was found, that is, the shift of DCJTB emission spectra with different hosts under the same concentration. Figure 6 illuminates the EL spectra of Device B and D. It is obvious that the EL spectrum of Device B occur blue-shift compared to Device D. Zhou and co-workers explained that the spectral blue-shift was derived from an incomplete energy transfer from exciplex to fluorescent emitter and the single emission peak was the overlap between the emission bands of exciplex and DCJTB^30^. But this explain is not agreed in this work. To clarify our viewpoint, we fabricated another device with the structure of ITO/MoO_3_ (3 nm)/NPB (20 nm)/TCTA (8 nm)/TCTA:TPBi (1:1): 1.0% DCJTB (15 nm)/TPBi (45 nm)/LiF (1 nm)/Al, which is referred as Device E. The EL emission peaks of TCTA:TPBi exciplex and Alq: 1.0% DCJTB locate at 460 and 618 nm, respectively, which could be divisive easily. (See the inset of Fig. 7). Thus, the EL spectra of Device E with 1.0% DCJTB doped into TCTA:TPBi as the EML would not occur the spectral overlap between the emission bands of exciplex and DCJTB. Figure 7 shows the EL spectra of Device E under different voltages. Under a low voltage, there is only single emission peak lying at 606 nm, which is the emission of DCJTB. As the voltage increases to 9 V, the exciplex emission peak of TCTA:TPBi appears, but 606 nm main peak still exists and do not occur shift. This prove that the single emission peak from device with TCTA:3P-T2T: 1.0% DCJTB as the EML is not the overlap between the emission band of exciplex and DCJTB.

Summarizing all the spectral shift mentioned above, include the spectral red-shift as the increase of DCJTB concentration under PL and EL and the spectral difference with different hosts under the same DCJTB concentration, we consider that the spectral shift is result from the molecular polarization effects^31^,^32^. Under the PL and EL, as the DCJTB concentration increases, the distance between DCJTB molecules decreases, which increases the local polarization field and the increased local polarization field tends to cause the spectral red-shift. On the other hand, we think the spectral shift is consistent with the dielectric constant of the film and the bigger dielectric constant, the more red-shifted spectra^32^. Under PL and EL, the dielectric constant should increase with the increased concentrations, which lead to the spectral red-shift. And the dielectric constants may also different under the doped film of TCTA:3P-T2T: 1.0% DCJTB and Alq: 1.0% DCJTB though they have the same doped concentration. Compared to the film of Alq: 1.0% DCJTB, TCTA:3P-T2T: 1.0% DCJTB should have a smaller dielectric constant, so a blue-shifted spectra could happen.

In conclusion, highly efficient DCJTB emission based on the energy transfer from delayed fluorescent exciplex host to fluorescent dopant was achieved. At the concentration of 1.0% DCJTB, we achieved a maximum luminance, current efficiency, power efficiency and EQE of 22,767 cd m^−2^, 22.7 cd A^−1^, 21.5 lm W^−1^ and 10.15%, respectively. Achievement of so high EL efficiency is ascribed to the application of delayed fluorescent exciplex host. That is, the exciplex singlet excitons and up-converted triplet excitons are transferred efficiently to singlet state of fluorescent DCJTB emitter. We find that the doped concentration plays a key role in achieving high EL performance. Our finding provides a new design way to achieve highly efficient fluorescent OLEDs with traditional fluorescent materials. Furthermore, it is believed that by exploitation of more efficient delayed fluorescent exciplex, higher efficiency OLEDs could be realized.

## Methods

### Sample preparation and characterization for photoluminescence

Samples for the optical measurements were fabricated by co-depositing exciplex host (TCTA:3P-T2T) and x% DCJTB (0, 0.5, 1.0 and 1.5) with a thickness of 40 nm on the glass substrate. The photoluminescence (PL) spectra were measured with F7000 spectrophotometer. Transient PL decay was measured with the combination of Nd-YAG laser (excitation wavelength of 355 nm, pulse width of 10 ns and repetition frequency of 10 Hz), spectrograph (HJY, Triax550) and oscilloscope (Tektronix, TDS3052). The photoluminescence quantum yield (PLQY) was measured with F900 fluorescence spectrometer (Edinburgh Instruments Ltd) and the system combined with an integrating sphere, a Xe lamp (as the excitation source) and a multichannel spectrometer (as the optical detector). Sample for the absorption measurement was fabricated by depositing neat DCJTB with a thickness of 20 nm on the quartz substrate. Absorption spectrum was measured with UV/VIS/NIR scanning spectrophotometer (Shimadzu, UV/3101PC).

### Sample preparation and characterization for electroluminescence

All the devices were fabricated on Indium tin oxide (ITO) coated glass substrates with a sheet resistance of 10 Ω/sq. The ITO substrates were cleaned with acetone, deionized water and acetone and then treated by ultraviolet-ozone for 15 min before loading into a high vacuum chamber (approximately 3 × 10^−4^ Pa). After the deposition of the organic layers, Al cathode was deposited finally with a shadow mask that defined an active device area of 3 × 3 mm^2^. The EL spectra were measured with OPT-2000 spectrophotometer. The current-voltage-luminance characteristics were measured with a Keithley model 2400 power supply combined with a ST-900M spot photometer and were recorded simultaneously with measurements. EQE was calculated from the current density-voltage-luminance curve and spectrum data.

All the organic materials for fabrication were procured commercially without further purification. All the measurements were carried out at room temperature and under ambient conditions without any protective coatings.

## Additional Information

**How to cite this article**: Zhao, B. *et al.* Highly efficient red OLEDs using DCJTB as the dopant and delayed fluorescent exciplex as the host. *Sci. Rep.* **5**, 10697; doi: 10.1038/srep10697 (2015).

## Figures and Tables

**Figure 1:**
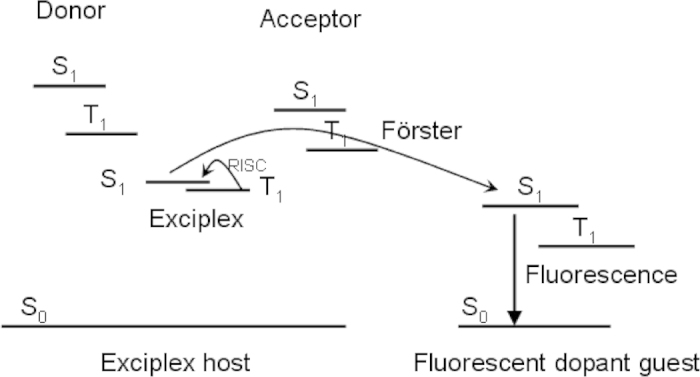
The schematic diagram and supposed energy transfer processes.

**Figure 2:**
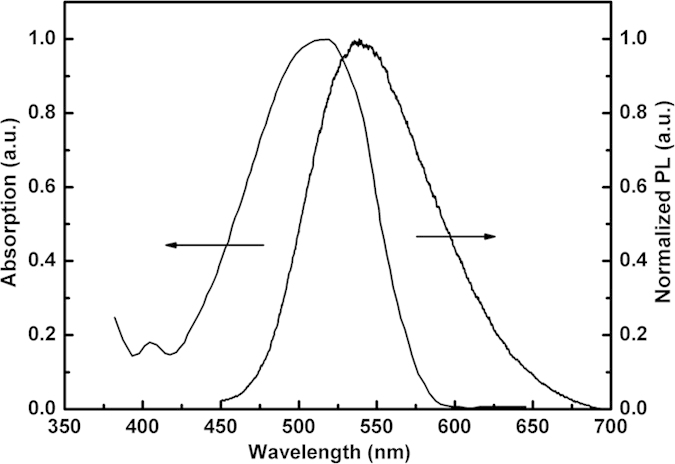
The normalized absorption spectrum of DCJTB and PL spectrum of co-deposited film of TCTA:3P-T2T (1:1).

**Figure 3:**
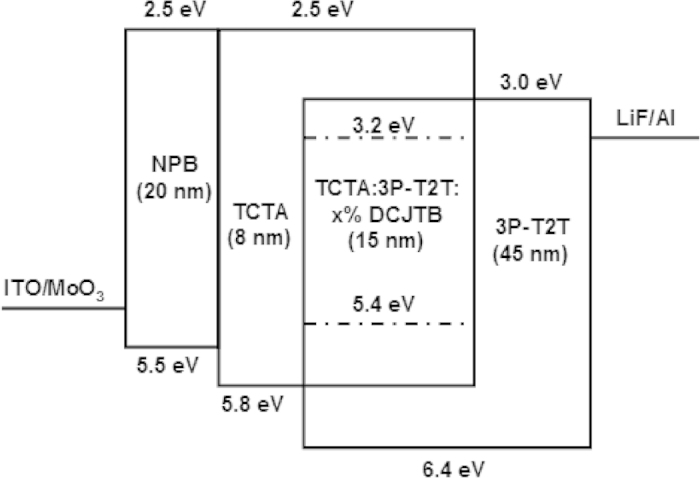
The energy level diagram of the red OLEDs with delayed fluorescent exciplex (TCTA:3P-T2T) as the host.

**Figure 4:**
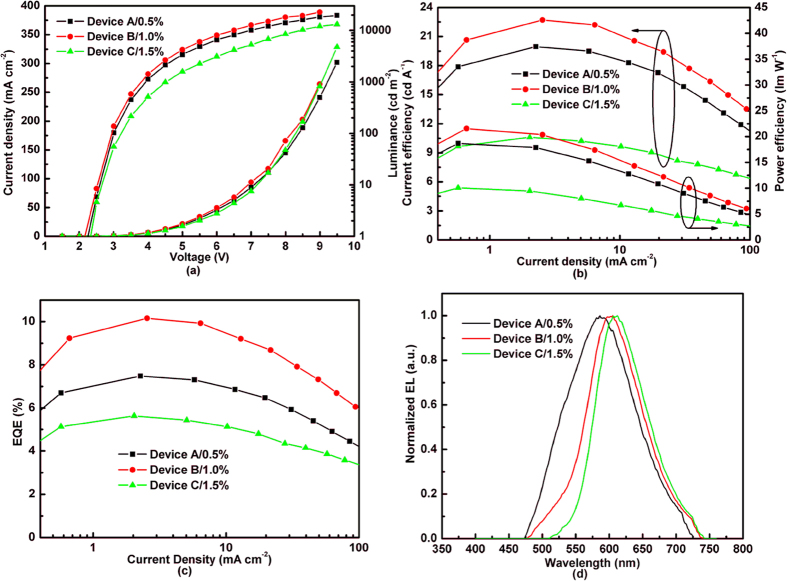
The EL characteristics curves with current density, luminance, current efficiency, power efficiency, EQE and EL spectra of Device A, B and C.

**Figure 5:**
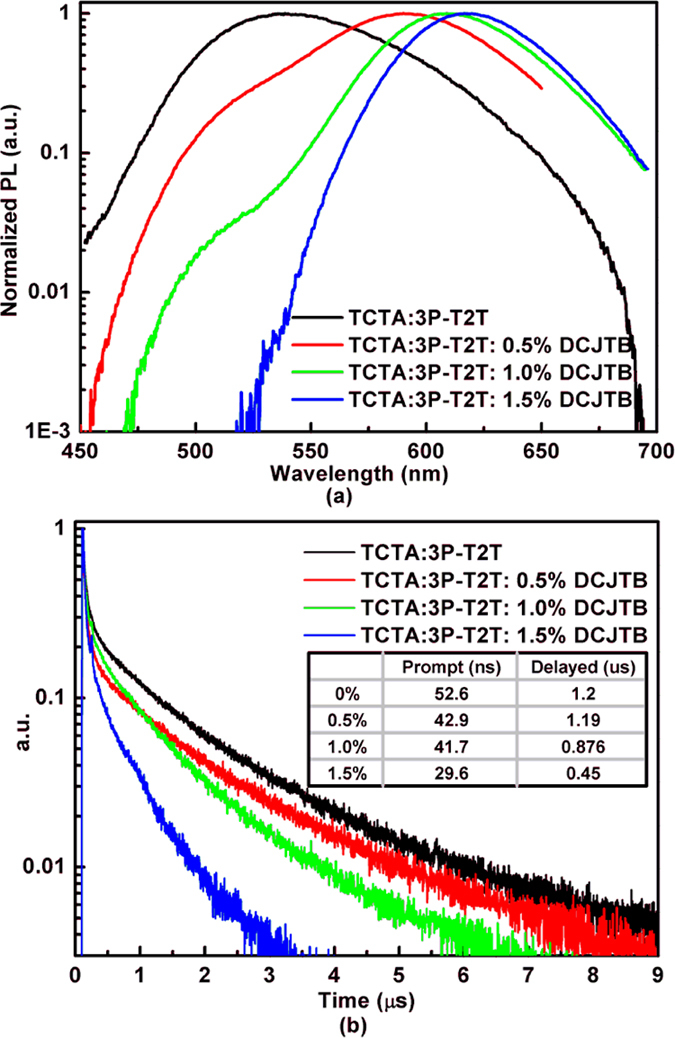
The PL spectra and transient PL decay curves of TCTA:3P-T2T and DCJTB doped into TCTA:3P-T2T with various concentrations (0.5%, 1% and 1.5%).

**Figure 6:**
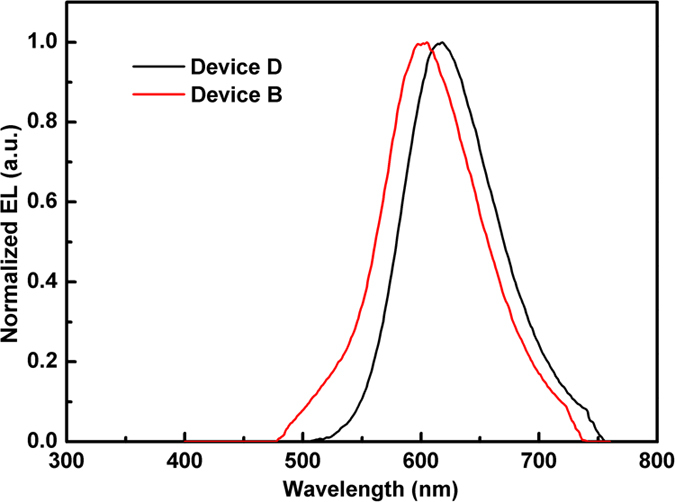
The EL spectra of Device B and D.

**Figure 7:**
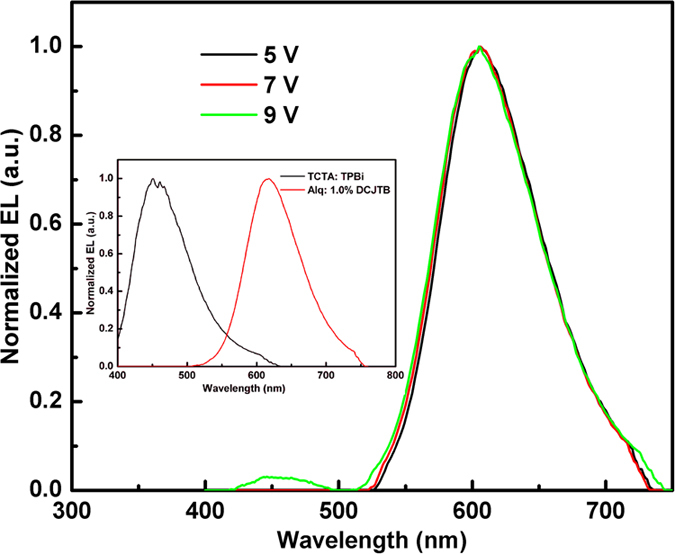
The EL spectra of Device E under different voltages. (Inset shows the EL spectra of TCTA:TPBi and Alq: 1.0% DCJTB as the EML, respectively).

**Table 1:** The summary EL data of Device A, B and C in this paper.

	V_on_^a^)	η_c,Max._/η_p,Max._/EQE_Max._^b^)	η_c,1000_/η_p1000_/EQE_1000_^c^)	η_c,10000_/η_p10000_/EQE_10000_^d^)
	[V]	[cd A^−1^/lm W^−1^/%]	[cd A^−1^/lm W^−1^/%]	[cd A^−1^/lm W^−1^/%]
Device A	2.25	19.9/18.6/7.48	19.6/15.8/7.32	11.9/5.4/4.51
Device B	2.17	22.7/21.5/10.15	22.4/18.9/10.03	15.0/7.3/6.72
Device C	2.33	10.5/10.1/5.63	9.6/6.7/5.13	5.3/2.8/1.95

^a)^Turn on voltage (V) at 1 cd m^−2^;

^b)^Current efficiency (η_c_), power efficiency (η_p_) and external quantum efficiency (EQE) at Maximum;

^c)^η_c_, ηp and EQE at 1000 cd m^−2^;

^d)^η_c_, ηp and EQE at 10000 cd m^−2^.
